# Can Wrist-Worn Medical Devices Correctly Identify Ovulation?

**DOI:** 10.3390/s23249730

**Published:** 2023-12-09

**Authors:** Angela Niggli, Martina Rothenbühler, Maike Sachs, Brigitte Leeners

**Affiliations:** 1 Department of Reproductive Endocrinology, University Hospital of Zürich, Frauenklinikstrasse 10, 8091 Zürich, Switzerland; maike.sachs@usz.ch (M.S.); brigitte.leeners@usz.ch (B.L.); 2 Faculty of Medicine, University of Zurich, 8032 Zurich, Switzerland; 3 Ava AG, Gutstrasse 73, 8055 Zürich, Switzerland

**Keywords:** fertility, menstrual cycle, ovulation, wearable, sensor

## Abstract

(1) Background: Hormonal fluctuations across the menstrual cycle lead to multiple changes in physiological parameters such as body temperature, cardiovascular function, respiratory rate and perfusion. Electronic wearables analyzing those parameters might present a convenient alternative to urinary ovulation tests for predicting the fertile window. (2) Methods: We conducted a prospective observational study including women aged 18–45 years without current hormonal therapy who used a wrist-worn medical device and urinary ovulation tests for a minimum of three cycles. We analyzed the accuracy of both the retrospective and prospective algorithms using a generalized linear mixed-effects model. The findings were compared to real-world data from bracelet users who also reported urinary ovulation tests. (3) Results: A total of 61 study participants contributing 205 cycles and 6081 real-life cycles from 3268 bracelet users were included in the analysis. The mean error in identifying ovulation with the wrist-worn medical device retrospective algorithm in the clinical study was 0.31 days (95% CI −0.13 to 0.75). The retrospective algorithm identified 75.4% of fertile days, and the prospective algorithm identified 73.8% of fertile days correctly within the pre-specified equivalence limits (±2 days). The quality of the retrospective algorithm in the clinical study could be confirmed by real-world data. (4) Conclusion: Our data indicate that wearable sensors may be used to accurately detect the periovulatory period.

## 1. Introduction

Approximately 15% of women of reproductive age are trying to conceive [1]. Difficulties in timing sexual intercourse to align with the fertile window, which is defined as the day of ovulation and the 5 preceding days, is a major factor in preventing pregnancy-seeking couples from conceiving [2]. The majority of women do not know about the period when they have the highest chances to conceive during their menstrual cycles [3]. Properly timed intercourse during the fertile window has been shown to increase the probability of conception in comparison to intercourse without ovulation prediction [4]. There is a clear medical need to help women identify their fertile windows in an accurate manner to better time intercourse and increase their chances of conceiving.

To date, ultrasound examination has served as the gold standard for ovulation detection; however, if performed correctly at least once per day, this method is not only costly but also inconvenient and therefore inappropriate to be used for long-term monitoring [5]. Alternatively, urine-based Luteinizing Hormone (LH) kits, which detect the LH surge [6,7,8,9] occurring 24–36 h prior to ovulation, [2,10] have been correlated with ovulation as detected by ultrasonography [6,7]. Although accurately heralding ovulation for most women [7,8,9], urinary LH tests prospectively identify only part of the fertile window prior to ovulation. Fertility awareness-based methods (FABMs) represent reliable alternatives, but they require sufficient education for the correct application of use, with their success dependent on user motivation and compliance [11].

Advances in mobile phone technology have led to the creation of apps designed for menstrual cycle tracking, simplifying and facilitating access to FABM methods. Those relying on calendarbased FABM methods fail to accommodate natural variances between and within a woman’s cycle (e.g., longer follicular phases due to temporary stress), leading to greater inaccuracy and sometimes even a counter-productive effect on conception chances, as they do not correctly predict the fertile window, and couples consequently aim for a pregnancy at the wrong time [2,10]. More recently, new technology including wearable sensors has demonstrated that measuring multiple physiological parameters has the potential to utilize FABM methods more accurately than methods that utilize only postovulatory shifts in the BBT [2,5,12]. Most of the current fertility tracking devices or mobile phone applications combine mechanisms of calendar cycle tracking, cervical mucus interpretation, urinary LH testing and/or BBT [13,14,15]. As a consequence, they necessitate multiple active diagnostic measures by the user, which result in an effort that is comparable to the previously available FABM methods. In addition, current FABM methods are often based on one singular daily measurement of, for example, the BBT. Wearable sensors enable automatic, continuous and simultaneous measurements, providing millions of data points of different physiological cycle-related parameters, and therefore offer a far more differentiated monitoring of the menstrual cycle [16]. They are established in body temperature surveillance; however, since the largest body temperature increase occurs postovulatory, the prediction of ovulation is not possible based on these data alone, and it requires additional clinical context [14,16]. Only the inclusion of physiological changes before ovulation allows for the reliable prediction of ovulation.

Changes in multiple physiological parameters due to hormonal fluctuations across the menstrual cycle have been well documented. Spontaneously menstruating women show measurable, natural variations not only in their body temperatures [17], but also in their cardiovascular function [18,19,20], bioimpedance [21,22], respiratory rates [23,24], and perfusion [25,26] depending on their cycle phases. To document these effects, most prior research has required hospital-grade medical equipment. In contrast, wearable technology provides a convenient tool that can correlate with changes in the physiological parameters and phases of the menstrual cycle [11,27]. As wearable technology is non-invasive, the continuous monitoring of multiple parameters simultaneously is also feasible and can deliver large datasets with a low burden for the user. The use of several simultaneously measured parameters, which all show specific patterns throughout the menstrual cycle, increases the predictive quality and, consequently, the reliability of the prediction [27]. Therefore, wearable technology has several important advantages which can not only improve the chances for pregnancy in a private or clinical setting, but can also monitor the menstrual cycle and ovulation for scientific purposes. While the methods that are currently available are only suitable for a certain subgroup of women who have a strong motivation to deal intensively with and understand the physical changes in their cycles, an innovative wearable device would also open up the possibility of cycle monitoring for users who do not want to or cannot make this effort.

In this study, we aimed to demonstrate that the performance of a wrist-worn medical device analyzing temperature and multiple other physiological parameters, which are not included in other fertility tracking devices, was equivalent to LH tests for the identification of ovulation. In addition, the known fluctuations in those parameters allowed us to design a real-time prospective ovulation prediction algorithm, and we compared these findings with results from a large real-world sample of bracelet users.

## 2. Materials and Methods

### 2.1. Study Design

This study compared the results from an LH urine test to predict ovulation using a wrist-worn medical device in a defined sample of healthy women of reproductive age who were not trying to conceive. These findings were compared to real-world data from bracelet users who also performed and reported urinary LH tests.

### 2.2. Study Population and Eligibility Criteria

Inclusion criteria for study participation were women, aged 18–45 years, free of any current hormonal therapy for at least 2 months, willingness to participate for at least 3 cycles in the study and not planning any pregnancy in the subsequent 3 months. For both study participants and real-world bracelet users, we only included regular cycles (24 to 35 days) with at least one positive LH test result, and at least 70% of the cycle days synced correctly and with luteal length between 9 and 20 days to reflect the clinical situation, i.e., control for signs of an eventual unplanned pregnancy when bleeding did not occur after the normal length of about 13 days.

The exclusion criteria for study participation were problems wearing the medical device or difficulties understanding the study procedure, any health-related issues that could affect the menstrual cycle, any consumption of medication or other substances that could affect the menstrual cycle or any of the physiological parameters investigated, frequent travel between time zones, sleeping disorders, sleeping less than 4 h per night or current breastfeeding. All participants gave their written consent to participate in the study.

As the bracelet is on the market to support conceiving efforts, users either aim for pregnancy or use the device to monitor their cycles. Some of the women also use urinary LH tests in parallel, which can be logged in the bracelet app on a voluntary basis. The real-world dataset consists of cycles collected between January 2019 and November 2020 using the same hardware version as in the clinical trial, irrespective of the women’s ages, and it is presented in Table 1.

### 2.3. Measurements

For the underlying analysis, study participants used a wrist-worn medical device (Ava Fertility Tracker, Ava AG, Zurich, Switzerland) and a urinary LH test (Clearblue Digital Ovulation test, SPD Swiss Precision Diagnostics GmbH, Geneva, Switzerland) for the duration of a minimum of 3 and a maximum of 6 completed menstrual cycles. For the real-world data, every cycle that fulfilled the selection criteria was evaluated.

The Ava Fertility Tracker is a non-invasive device intended to measure and display physiological parameters to aid women in ovulation identification to facilitate conception.

The wrist-worn medical device works by identifying changes in multiple physiological parameters, including the wrist skin temperature (WST), heart rate, heart rate variability (HRV), respiratory rate and skin perfusion, based on an algorithm utilizing data from prior cycles and features extracted from the physiological parameter changes [11,27].

The electronic wearable device saves physiological information captured by temperature sensors, an accelerometer and a photoplethysmography every 10 s automatically. Participants were instructed to wear the bracelets nightly while sleeping on their wrists and to sync the bracelets with the complementary app on their smartphones each morning upon waking.

Based on data from a prospective cohort study of 237 conception-seeking women, techniques from machine learning were used to develop an algorithm for predicting and detecting a woman’s fertile window in real time [27]. To avoid variation induced by the initial drop in body temperature at the onset of sleep and the subsequent rise prior to waking, as well as perturbances of the other cycle-related parameters measured by the wearable device [28], the first 90 min and the last 30 min of each night’s data were excluded.

We used a cycle-based, random 75:25 split for the training and testing datasets, with each user belonging to only 1 category. The initial training dataset consisted of physiological observations from 186 users across 499 cycles, whereas the validation dataset initially contained data from 51 users across 166 cycles. We then trained a random forest model with 1000 trees and a max feature parameter of 3 on the training dataset using the Python module sklearn.ensemble. RandomForestClassifier [29] was used, and the setting of max_features = 3. We provided 11 input features engineered from the base physiological signals including the heart rate, breathing rate, WST and HRV. We used follicular phase, fertile window with the day of ovulation and luteal phase for classification and kept all cycles in our training dataset. For cycles where participants had synced their data nightly at least 80% of the time, our model used those features in estimating the fertile window. For cycles where nightly data were synced less than 80% of the time, however, the algorithm instead predicted the upcoming fertile window based on the user’s previous cycle length and length of their typical luteal phase. Following the fertility algorithm’s training, we tested it using the validation dataset to determine its performance [27].

The algorithm prospectively identifies the opening and closing of the fertile window as well as ovulation. The wrist-worn medical device is registered as a Class 1 medical device with Swissmedic. Participants tracked and reported their ovulation each cycle using the urinary ovulation test on pre-specified days of their cycle. Luteal phase was defined as the day after ovulation until and including the day preceding the day of onset of the next menstrual period.

The urinary ovulation test was the standard reference in this trial. Participants were asked to report their LH results in the respective dedicated field of their Ava applications.

### 2.4. Outcomes

The primary outcome of the study was the error in the retrospective (i.e., after the end of a cycle) detection ovulation day of the wrist-worn medical device compared to the reference LH test. The difference in determining ovulation day between the index and the reference test is expressed in number of days.

The first secondary outcome consists of the error in days in prospectively detecting the ovulation day of the wrist-worn medical device compared to the reference test. Other secondary outcomes are the sensitivity, specificity and accuracy of both the retrospective and prospective algorithms of the wrist-worn medical device as well as a comparison of the results in the defined study group with the real-life data. True positive days are defined as correctly identified fertile days, in that both the index and reference tests indicate the days as fertile (i.e., within the fertile window), and true negative days are defined as correctly identified infertile days, in that both the index and reference tests indicate the days as infertile (i.e., outside the fertile window).

### 2.5. Sample Size Calculation

The primary objective of this prospective diagnostic accuracy study was to estimate whether the wrist-worn medical device index test is equivalent to the reference test in detecting ovulation. Ovulation was considered to happen 24 h after an LH surge. The sample size estimation considered a two one-sided test procedure for a one-sample equivalence test with an expected mean difference of zero [30,31,32], an alpha of 0.025 and a statistical power of 90%. Furthermore, we assumed a clinically meaningful margin of ±2 days and a standard deviation of 3 days. The clinically meaningful margin of ±2 days was justified by the daily conception probabilities during the fertile window. In case the wrist-worn medical device index test detected ovulation day with an error of two days, the user still had four days of the fertile window. In cases when the wrist-worn medical device index test detected ovulation two days too early, the participant would have four days to have conceptive intercourse, with conception probability ranging between 0.08 and 0.34 [4]; a shift of two days later would have shown that the user had four days, with conception probability between 0.08 and 0.36 [4].

(1) $$n_{L}=\frac{(z_{\alpha }+z_{\beta /2}{)}^{2}\sigma^{2}}{\delta_{L}^{2}},\;n_{U}=\frac{(z_{\alpha }+z_{\beta /2}{)}^{2}\sigma^{2}}{\delta_{U}^{2}}$$

Equation (1): Sample size calculation using two one-sided equivalence tests.

Since we adopted two one-sided test procedures with symmetric equivalence limits around zero, the respective estimated sample sizes for the clinically meaningful lower margin ($n_{L}$) and for the clinically meaningful upper margin $\left(n_{U}\right)$ are identical [33]. As the unit of analysis is cycles clustered within women, the required sample size was multiplied by the design effect, which consists of both the intra-cluster correlation coefficient (ICC) that characterizes the correlation of cycles within women and the number of cycles per woman. We estimated the ICC in terms of the cycle length using data from another trial of the wrist-worn medical device of a similar design (NCT03161873).

Assuming a clinically meaningful margin of ±2 days, a standard deviation of 3 days, a mean difference of zero and an ICC of 0.147, and considering that the number of cycles per woman is 3, we required 39 cycles from 13 women to detect equivalence in identifying ovulation with 90% power and a one-sided alpha of 0.025.

The assessment of the primary objective required that information regarding the reference test was available for each cycle and that at least 70% of cycle days of the index test were synced. We assumed that up to 50% of the cycles were affected by data quality issues such as insufficient data syncs of the wrist-worn medical device index test, missing ovulation data because of anovulatory cycles or forgotten LH reference tests. Furthermore, by accounting for a 20% loss to follow up in participants for each completed cycle, we estimated a need to recruit a total of 58 women.

For the evaluation of real-life data, we included any cycle that met the inclusion criteria.

### 2.6. Statistical Analysis

Participants are described with respect to age, BMI, ethnicity and time since stopping hormonal contraception. Categorical data are summarized by counts and percentages. Continuous data are summarized by mean and standard deviation in case of normally distributed data. Cycle characteristics such as mean cycle length, mean luteal length or mean number of cycles per women are reported.

The analysis of the primary outcome includes all cycles with a positive LH test result and a bracelet syncing rate of ≥70%. We performed a retrospective analysis (retrospective algorithm) of the wrist-worn medical device compared to the LH test. The retrospective algorithm is used for the determination of the fertile window at the end of the menstrual cycle, while the prospective algorithm is used to define the fertile window in a live setting, as the physiological data are being collected, which is why we cannot provide these data for the real-life setting, where the data could only be analyzed retrospectively.

The primary outcome was assessed using a generalized linear mixed-effects model to account for the fact that cycles are nested within women. We used the R package glmmTMB [33] to analyze the primary outcome, which is the error in the identification of ovulation compared to the urinary LH reference test. We specified a generalized linear mixed model with random effects to allow for random variation in slopes through the sequence of cycles across participants and with an autoregressive structured variance–covariance matrix. We evaluated both the distribution of the residuals and model convergence. The sequence of cycles across women was defined independently of compliance or protocol deviations to account for the fact that the algorithm of the wrist-worn medical device learns from past cycles. So, for some women, the sequence of cycles was not increasing with an interval of 1, and others had a missing first cycle. Equivalence was declared if the lower limit of the two-sided 95% confidence interval of the mean error was not lower than the pre-specified clinically meaningful lower limit, and if the upper limit of the two-sided 95% confidence interval of the mean error was not higher than the pre-specified clinically meaningful upper limit. Correspondingly, the one-sided α-level was 0.025.

We estimated and compared the sensitivity, specificity and accuracy of both the retrospective and prospective algorithms, accounting for the correlation within observations both at the cycle and participant levels. Finally, we compared the error in detecting ovulation, the sensitivity, specificity and accuracy of the retrospective algorithm using the real-world population.

### 2.7. Ethics

The study was approved by the cantonal ethics committee of the canton of Zurich (BASEC Nr PB_2016-2670). Women provided written informed consent on study participation as well as on the use of their data for scientific evaluations.

## 3. Results

### Study Population

**Figure 1:**
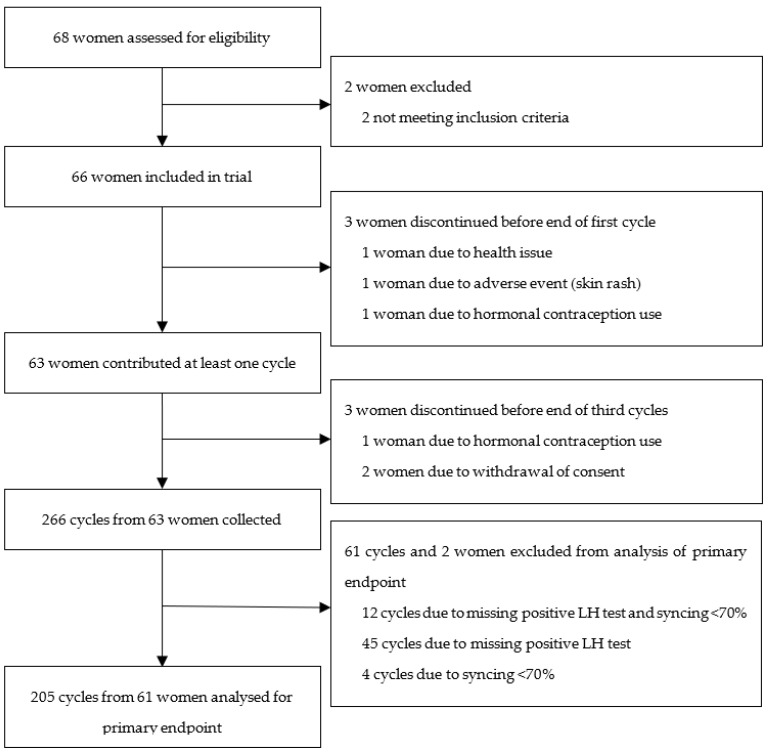
Recruitment of study participants.

Figure 1 shows the recruitment of the study population. Of the 66 participants included in the trial, 61 participants, contributing 205 cycles, were included in the analysis of the primary outcome. Three participants were excluded from the analysis due to discontinuation before the end of their first completed cycle. Of these participants, one experienced an adverse event (skin rash), one participant reported no longer having her period and the remaining participant switched to hormonal contraception during her first cycle. Three participants discontinued before the end of the regular study. An additional 61 cycles were excluded due to missing LH test results and/or due to a syncing rate of the wrist-worn medical device of <70%.

In addition to the 205 cycles of the study participants, a total of 6081 real-life cycles from 3268 bracelet users fulfilled the criteria for being included in the analysis.

The socio-demographic data of women participating in the clinical trial and the real-world data are presented in Table 1.

The mean age of the 66 participants included in the analysis of the clinical study was 26.5 years (SD ± 4.2), and the mean age of the women who provided real-world data was 32.5 years (SD ± 4.3). The mean BMI values were 22.3 (±2.9) and 25.5 (±6.27)., respectively. In the participants of the clinical trial, most of the participants (77%) were white, as were most of the women who provided the real-world data (80.3%). The participants of the clinical trial wore the Ava Fertility Tracker for 3.36 (±1.18) cycles, on average, while the real-world users provided an average of 1.86 (±1.28) cycles per woman.

Table 2 provides an overview of the cycle characteristics in both study groups. The participants of the clinical trial had a mean cycle length of 29.6 (±3.2) days, and the women who provided the real-world data had a mean cycle length of 28.5 (±2.8), and the mean luteal lengths were 12.7 (±0.8) and 12.6 (±0.7), respectively.

The primary analysis included 61 women who participated in the clinical trial and contributed 205 cycles. The quality markers of the algorithms are summarized in Table 3. Of the 205 cycles, 57 cycles (27.8%) identified ovulation with an error of zero days, and 168 cycles (82%) identified ovulation within the pre-specified equivalence limits (±2 days). Of the cycles with errors outside of the equivalence limits, the Ava retrospective algorithm identified ovulation after the LH reference test in 26 cycles (12.7%), and before the LH references test in 11 cycles (5.4%). The prospective algorithm of the Ava Fertility Tracker identified ovulation in 36 cycles (17.6%) with a mean error of zero days, with a total of 148 cycles (72.2%) being within the pre-defined confidence limit.

The overall performance of the retrospective algorithm was slightly higher than that of the prospective algorithm. Over three quarters of all fertile days (75.4%) were correctly labeled by the retrospective algorithm, and 73.8% was correctly labeled by the prospective algorithm. For the retrospective algorithm in the clinical trials, the accuracy, sensitivity and specificity were 0.93 (95% CI 0.91 to 0.94), 0.80 (95% CI 0.76 to 0.83) and 0.95 (95% CI 0.94 to 0.95), respectively, and for the prospective algorithm, the values were 0.89 (95% CI 0.87 to 0.90), 0.77 (95% CI 0.71 to 0.82) and 0.91 (95% CI 0.90 to 0.92).

**Figure 2:**
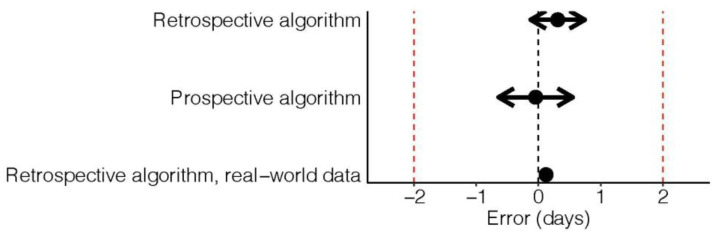
Mean error in identification of ovulation.

The real-world data showed an accuracy, sensitivity and specificity of 0.91 (95% CI 0.91 to 0.92), 0.77 (95% CI 0.76 to 0.78) and 0.94 (95% CI 0.93 to 0.94), respectively. Altogether, 74.6% of all fertile days were allocated correctly. Figure 2 gives an overview of the precision of each algorithm.

The mean error in detecting ovulation with the retrospective algorithm was 0.31 days (95% CI −0.13 to 0.75) and -0.04 days with the prospective algorithm (95% CI −0.64 to 0.55), both with a *p*-value for equivalence of <0.001. The referring results for the retrospective algorithm in the real-world data were 0.12 days, (95% CI 0.06 to 0.18) and *p* < 0.0001.

## 4. Discussion and Conclusions

With this publication, we provide evidence from a head-to-head, prospective, diagnostic accuracy study for the investigated wrist-worn medical device equivalence in detecting ovulation with a urine-based LH surge measurement that occurs 24–36 h prior to ovulation [2,10]. Second only to an ultrasound examination, an LH measurement serves in most studies as the gold standard for ovulation detection, as a serial ultrasound is costly, inconvenient and inappropriate for home monitoring of the menstrual cycle [5,6,7]. In the predefined primary study outcome, the mean error in identifying the LH-defined ovulation with the wrist-worn medical device retrospective algorithm was 0.31 days (95% CI −0.13 to 0.75). This error was well within the predefined margin of error of ±2 days, with a p-value for equivalence of <0.001. As we were particularly strict and considered ovulation as the LH surge +24 h instead of the otherwise tolerated + 36hours, we overestimated our error. As the mean error in detecting ovulation was even less in the real-world data at 0.12 days (95% CI 0.06 to 0.18, *p* < 0.0001), this study demonstrates that the wrist-worn medical device was equivalent to LH in identifying ovulation.

Also, the wrist-worn medical device’s prospective algorithm mean error in detecting ovulation was only −0.4 days (95% CI −0.64 to 0.55) and well within the predefined error margin for equivalence to the LH. The clinically meaningful margin of ±2 days was justified by the high daily conception probabilities during the entire fertile window. For cases where the wrist-worn medical device index test detects the ovulation day with an error of two days, the user would still have four fertile window days to conceive. It is important to note that because LH tests prospectively identify only the part of the fertile window shortly prior to ovulation, the wrist-worn wearable device can flag more days that are suitable for conceptive intercourse as it may additionally identify more potentially fertile days early in the fertile window in real time. It is also more convenient than repeat urine testing.

The findings in this study are consistent with previous research conducted on the wrist-worn medical device [11,27,32]. In those studies, we found that the device can identify significant, concurrent phase-based shifts in the wrist skin temperature (WST), heart rate and respiratory rate that are robust to daily, individual and cycle-level covariates. We found significantly higher resting pulses and respiratory rates during the users’ luteal phases compared with their menstrual phases. Those changes combined with post ovulatory biphasic shifts in the WST can be used to identify the opening and closing of their fertile windows. In the present study, the wrist-worn medical device’s machine learning algorithm can detect part of a user’s fertile window with an 89% accuracy in real time (95% CI 0.87 to 0.90).

The assessment of the primary objective required that an LH measurement be available for each cycle and that the user synced the Ava Fertility Tracker on at least 70% of cycle days. Thus, in the group of participants of the clinical trial, a total of 61 cycles (23%) were excluded from the analysis due to missing LH test results and/or a low syncing rate of the wrist-worn medical device. This was considered acceptable, given that many studies report anovulation in 10 to 20% of cycles [34,35].

The performances of both the retrospective algorithm as well as the real-time prospective algorithm to detect ovulation were also favorable. The overall performance of the retrospective algorithm was slightly higher than that of the prospective algorithm. Over three quarters of all fertile days (75.4%) were correctly labeled by the retrospective algorithm, and 73.8% were correctly labeled by the prospective algorithm. The accuracy of the wrist-worn medical device’s retrospective algorithm was 0.93 (95% CI 0.91 to 0.94), and for the prospective algorithm, it was 0.89 (95% CI 0.87 to 0.90), respectively. With a correct detection of 74.6% of the fertile days, as represented by the LH measurements, and an accuracy of 0.91 (95% CI 0.91 to 0.92), the findings from the retrospective algorithm in the study population were confirmed by our large sample of real-world cycles. Therefore, the algorithm does not only perform reliably under study conditions but also in real-world conditions.

Similar to the findings from studies using more traditional basal body temperature (BBT) methods for temperature tracking [2,25,36], we found a pattern of a significantly lower WST in the follicular and fertile phases and a significantly higher WST in the late luteal phase compared to menses [11,24]. However, the WST measured by the device identified more post ovulatory biphasic shifts (55%) correctly than the traditional BBT method (20%), and the WST that was continuously measured during sleep by the wearable bracelet was more sensitive than the BBT for detecting ovulation [37]. The continuous monitoring with wearable sensor technology has proven to be superior to single daily measurements [37].

### Strengths and Limitations

A strength of this study is its well-designed methodology, providing data from an adequately powered sample investigated within study conditions as well as from a large sample of real-word users. Although the LH test is the current the gold standard for convenient ovulation monitoring, this method is only a surrogate measure of ovulation and not fully reliable, which will have influenced our results. Unfortunately, our real-life data only allowed for the evaluation of our retrospective algorithm so that we could not draw any conclusions on the prospective performance in this sample.

## 5. Conclusions

Wearable sensor technology allows for the continuous and simultaneous measurement of several parameters, and this study demonstrates that it is possible to detect ovulation with this approach. Therefore, the wrist-worn medical device is well positioned to provide an alternative method to increase the chances for conception, and it is as accurate as the gold stand of urinary LH testing while being more simple and convenient than historic FABM and more holistic than other mainly temperature-based sensors.

## Figures and Tables

**Table 1:** Socio-demographic data of women participating in the clinical trial and real-world data provided by women.

	Clinical Trial Data	Real-World Data
Women	61	3268
Age (yrs)	26.5 (±4.2)	32.5 (±4.3)
Age (yrs) range	18 to 35	19 to 47
Age categories		
18–20	4 (6.6%)	2 (0.1%)
21–15	23 (37.7%)	137 (4.2%)
26–30	24 (39.3%)	896 (27.4%)
31–35	10 (16.4%)	1406 (43%)
36–40	0 (0%)	575 (17.6%)
41–45	0 (0%)	114 (3.5%)
>46	0 (0%)	8 (0.2%)
No answer	0 (0%)	130 (4%)
Height (cm)	166.3 (±6.1)	164.5 (±9.1)
Weight (kg)	61.2 (±8.5)	69.4 (±18.9)
BMI	22.1 (±2.9)	25.5 (±6.27)
BMI category		
Underweight	2 (3.3%)	97 (3%)
Normal	53 (86.9%)	1791 (54.8%)
Overweight	4 (6.6%)	762 (23.3%)
Obese	2 (3.3%)	618 (18.9%)
Ethnicity		
White	47 (77%)	2623 (80.3%)
Asian	3 (4.9%)	136 (4.2%)
Hispanic	4 (6.6%)	149 (4.6%)
Black	0 (0%)	83 (2.5%)
Other	7 (11.5%)	277 (8.5%)
Time since stopping hormonal contraception		
≤3 months	5 (8.2%)	351 (10.7%)
4–6 months	3 (4.9%)	313 (9.6%)
7–9 months	2 (3.3%)	315 (9.6%)
10–12 months	16 (26.2%)	246 (7.5%)
>12 months	19 (31.1%)	1912 (58.5%)
≥2 cycles without any further specification	16 (26.2%)	131 (4%)
Number of cycles per woman	3.36 (±1.18)	1.86 (±1.28)

**Table 2:** Description of cycles included in primary analysis.

	Clinical Trial Data	Real-World Data
Women included in analysis of primary outcome	61	3268
Cycles included in analysis of primary outcome	205	6081
Cycle length (95% CI)	29.56 (29.0 to 30.1)	28.54 (28.47 to 28.6)
Luteal length (95% CI)	12.67 (12.48 to 12.86)	12.61 (12.56 to 12.66)
Cycles outside of range of 24 to 35 days	11	
Cycle length (95% CI) among regular cycles (24 to 35 days)	28.95 (28.6 to 29.3)	
Retrospective algorithm		
Range of error to true ovulation	−18 to 10	−9 to 8
Range of error to true ovulation among regular cycles (24 to 35 days)	−5 to 10	
Cycles with errors outside of the lower equivalence limit (−2 days)	11 (5.4%)	534 (8.78%)
Cycles with errors outside of the upper equivalence limit (+2 days)	26 (12.7%)	684 (11.25%)
Cycles with error of zero	57 (27.8%)	1367 (22.48%)
Cycles within confidence limits	168 (82%)	4863 (79.97%)
Prospective algorithm		
Range of error to true ovulation	−7 to 25	
Range of error to true ovulation among regular cycles (24 to 35 days)	−7 to 12	
Cycles with errors outside of the lower equivalence limit (−2 days)	29 (14.2%)	
Cycles with errors outside of the upper equivalence limit (+2 days)	28 (13.7%)	
Cycles with error of zero	36 (17.6%)	
Cycles within confidence limits	148 (72.2%)	

**Table 3:** Sensitivity, specificity and accuracy in the detection and prediction of fertile days.

	Clinical Trial Data		Real-World Data
	Retrospective Algorithm	Prospective Algorithm	Retrospective Algorithm
True positive days *	927/1230 (75.4%)	908/1230 (73.8%)	27,205/36,486 (74.6%)
True negative days	4527/4830 (93.7%)	4383/4830 (90.8%)	126,911/136,192 (93.2%)
False positive days	303/4830 (6.3%)	447/4830 (9.3%)	9281/136,192 (6.8%)
False negative days	303/1230 (24.6%)	322/1230 (26.2%)	9281/36,486 (25.4%)
Sensitivity (95% CI)	0.80 (0.76 to 0.83)	0.77 (0.71 to 0.82)	0.77 (0.76 to 0.78)
Specificity (95% CI)	0.95 (0.94 to 0.95)	0.91 (0.90 to 0.92)	0.94 (0.93 to 0.94)
Accuracy (95% CI)	0.93 (0.91 to 0.94)	0.89 (0.87 to 0.90)	0.91 (0.91 to 0.92)
Mean error ** (days, 95% CI)	0.31 (−0.13 to 0.75)	−0.04 (−0.64 to 0.55)	0.12 (0.06 to 0.18)
*p*-value for equivalence	<0.0001	<0.0001	<0.0001

* Positive days = fertile days as represented by LH tests. ** Error in identifying ovulation compared to LH reference test.

## Data Availability

The data are available on request due to restrictions, e.g., privacy or ethical restrictions.
